# A transcriptome-wide Mendelian randomization study to uncover tissue-dependent regulatory mechanisms across the human phenome

**DOI:** 10.1038/s41467-019-13921-9

**Published:** 2020-01-10

**Authors:** Tom G. Richardson, Gibran Hemani, Tom R. Gaunt, Caroline L. Relton, George Davey Smith

**Affiliations:** MRC Integrative Epidemiology Unit (IEU), Population Health Sciences, Bristol Medical School, University of Bristol, Oakfield House, Oakfield Grove, Bristol, BS8 2BN UK

**Keywords:** Gene expression, Genetic association study, Quantitative trait, Medical research

## Abstract

Developing insight into tissue-specific transcriptional mechanisms can help improve our understanding of how genetic variants exert their effects on complex traits and disease. In this study, we apply the principles of Mendelian randomization to systematically evaluate transcriptome-wide associations between gene expression (across 48 different tissue types) and 395 complex traits. Our findings indicate that variants which influence gene expression levels in multiple tissues are more likely to influence multiple complex traits. Moreover, detailed investigations of our results highlight tissue-specific associations, drug validation opportunities, insight into the likely causal pathways for trait-associated variants and also implicate putative associations at loci yet to be implicated in disease susceptibility. Similar evaluations can be conducted at http://mrcieu.mrsoftware.org/Tissue_MR_atlas/.

## Abstract (web summary)

Gene expression and how genetic variants can influence gene expression are tissue-specific processes with important implications for phenotypes. Here, Richardson et al. use eQTL data from GTEx and the eQTLGen project in a two-sample SMR + HEIDI framework for causal inference of gene expression associations with complex trait.

## Introduction

Advancements in high-throughput sequencing technologies present an unparalleled opportunity to investigate the molecular determinants of complex disease. This has facilitated the identification of genetic variants that influence gene expression, known as expression quantitative trait loci (eQTL). Recent studies have demonstrated the benefit of using eQTL data to help understand the underlying mechanisms of findings from genome-wide association studies (GWAS)^1–3^. Moreover, endeavours leveraging eQTL data derived from different tissue types can help to further ascertain the biological and clinical relevance of variants associated with complex traits^4–6^. In particular, these efforts are important when investigating tissue specificity, the phenomenon whereby a gene’s function is restricted to particular tissue types^7^.

An important challenge in molecular epidemiology is assessing how associations between gene expression and complex traits depend upon the tissue analysed. We previously proposed an analytical pipeline to detect associations between tissue-specific gene expression and complex traits by applying the principles of Mendelian randomization (MR)^8–10^. This approach harnesses eQTL as instrumental variables (IVs) to investigate whether genetic variants at a locus influence both gene expression and complex trait variation. Furthermore, this framework has advantages over alternative transcriptome-wide approaches by incorporating techniques in genetic colocalization^11,12^. This helps to mitigate the likelihood of spurious findings attributed to two separate but correlated variants at a locus, one responsible for influencing gene expression and the other affecting the associated complex trait. As such, associations supported by evidence of genetic colocalization are more likely to be driven by a shared genetic factor. Crucially, we note that genetic colocalization is necessary, but not sufficient, for causality. This is because the genetic effect may influence the associated trait due to mediated changes in gene expression, or it may operate on both through independent biological pathways^13^.

In this study, we apply our framework to comprehensively evaluate the association between the transcription of 32,116 protein-coding, RNA- and pseudo genes, and 395 complex traits. To assess the importance of tissue dependency for these associations, we use gene expression from 48 tissue types using data from the genotype-tissue expression (GTEx) consortium^14^ (v7), as well as whole blood-derived data from the eQTLGen project^15^ (*n* = 31,684). With this putative causal map of tissue-dependent associations, we undertake several extensive analyses. Firstly, we evaluate the relationship between gene expression across many tissues and pleiotropy; the phenomenon whereby a gene influences variation in multiple traits^16^. Next, we undertake a series of transcriptome and phenome-wide analyses to uncover tissue-dependent associations. Findings such as these can help to develop insight into the underlying regulatory mechanisms that reside along the causal pathway from a genetic variant to its associated complex trait. Moreover, they can help uncover pleiotropic effects that may be confined to separate tissue types.

We also demonstrate that phenome-wide evaluations of target genes have translatable value. For example, they can help predict whether therapeutic intervention will result in potential on-target side effects, as well as propose scope for drug repurposing. This is particularly attractive as previous findings suggest that support from genetic association studies can improve efficacy and safety rates for drug validation efforts ^17,18^. Finally, we explore the tissue dependency of associations between selected genetic variants and blood pressure traits. Our findings suggest that integrating tissue-specific eQTL data can help prioritize likely functional genes and tissues responsible for GWAS signals.

## Results

### Constructing an atlas of tissue-dependent associations

We pooled together eQTL data from the GTEx consortium (v7) for 48 tissue types (*n* = 80–491, Supplementary Table 1) and the eQTLGen project using findings derived from whole blood (*n* = 31,684). Full summary statistics for 395 complex traits were obtained from large-scale GWAS (Supplementary Data 1). To investigate the association between the transcription of up to 32,116 genes (i.e., protein-coding, RNA- and pseudo genes) and each trait in turn, we applied two-sample summary MR^19^ and assessed genetic colocalization using the heterogeneity in dependent instruments (HEIDI) method (v0.710)^2,20^.

**Fig. 1:**
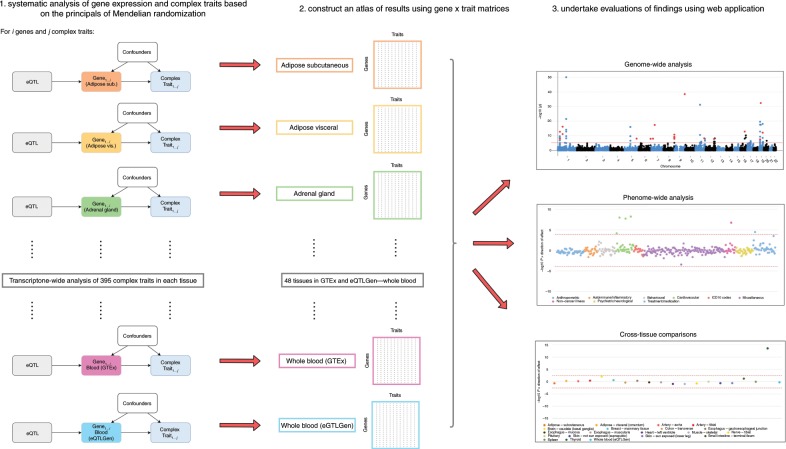
A schematic of the analysis plan in this study. An overview of the analysis pipeline applied in this study to assess the association between genetically predicted gene expression derived from 48 tissue types with 395 complex traits and diseases.

This approach was chosen over alternatives due to the vast majority of genes only having a single independent eQTL that can be used as an IV in an MR framework (based on *r*^2^ < 0.001) using tissue-specific data from GTEx. For example, only 285 of the 7865 genes with an eQTL using thyroid-derived gene expression have more than a single instrument. This caveat meant that we decided to undertake all analyses in our study using the top eQTL for each gene and applied the HEIDI method to reduce the likelihood of false-positive findings. Furthermore, knowing that our analyses were confined to using the top eQTL only, we also decided to apply a lenient *P* value threshold of *P* < 1.0 × 10^−04^ (based on linear regression coefficients) to define lead eQTL as IVs in our analysis. This was to include as many cross-tissue comparisons in our study as possible where results have all been derived using the same single IV approach. All findings can be visualized and downloaded using our web application located at http://mrcieu.mrsoftware.org/Tissue_MR_atlas/. A schematic of our study analysis can be found in Fig. 1.

Each analysis undertaken was adjusted for conventional genome-wide corrections (i.e., MR *P* < 5.0 × 10^−08^) and filtered for evidence of genetic colocalization (i.e., based on a HEIDI threshold of *P* > 0.05 to control for false-positive rates). In total, 39,586 MR associations were robust to multiple testing and genetic colocalization based on these criteria. Estimating *F*-statistics for these instruments used in these analyses suggested that eQTL with weaker evidence of association may reduce instrument strength as expected, although all instruments had an *F*-statistic > 10 (Supplementary Data 2). However, the *P* value threshold for eQTL instruments (*P* < 1.0 × 10^−04^ based on linear regression analyses) is simply a heuristic for highlighting associations worthy of follow-up^21^. Investigations of results can therefore apply more (or less) stringent lead eQTL and HEIDI thresholds by filtering associations downloadable from the web application.

We hypothesized that variants which influence gene expression levels in multiple tissues are more likely to influence multiple complex traits. To investigate this, we firstly grouped associations according to the organ that tissues were derived from (Supplementary Table 2). The reason for this is because we may expect similar association signals to be shared between tissues in GTEx which were part of the same embryonic tissue during development. For example, the various types of brain tissue from the GTEx consortium (e.g., amygdala, cerebellum, etc) were allocated to the ‘brain’ tissue group. This was to reduce false-positive findings from effectively counting the same association twice (e.g., gene expression in various types of brain tissue associated with the same neurological trait).

**Fig. 2:**
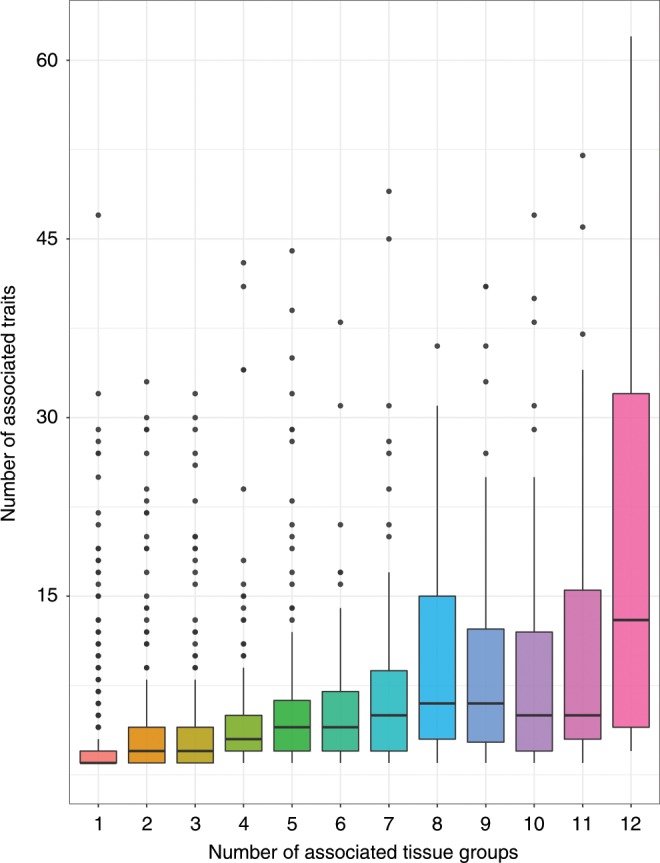
Box plot depicting the trend of gene expression in multiple tissues against pleiotropy. Box plot portraying the correlation in our atlas that genetically determined gene expression is more likely to be associated with multiple traits when expressed across multiple diverse tissue types. Whisker portray maximum and minimum values, whereas bounds of the boxes represent 25 and 75% quantiles and the centre lines the median values.

We identified strong evidence of a positive relationship between the number of associated traits for each lead eQTL and the number of tissues they were detected in (linear regression: beta = 1.14, s.e. = 0.03, *P* < 1.0 × 10^−16^). This analysis was adjusted for minor allele frequencies, linkage disequilibrium (LD) score, and distance to gene expression probe for lead eQTL, given that these genomic properties may influence the number of associated traits for a given SNP. In a subsequent analysis, we clustered eQTL effects based on their associated genes. Overall, there was a positive correlation between the number of traits that each gene was associated with and the number of different tissue groups that these associations were detected across (*r*^2^ = 0.46, Supplementary Fig. 1). As a sensitivity analysis, we determined tissue similarity by clustering based on Euclidean distance matrix computation (Supplementary Fig. 2). Repeating our analysis did not drastically change the identified positive correlation (*r*^2^ = 0.44, Fig. 2). This was also the case when repeating our analysis after clustering traits based on their subcategories and after excluding human leukocyte antigen (HLA) loci (both *r*^2^ = 0.42).

### A transcriptome-wide evaluation of thyroid disease

**Fig. 3:**
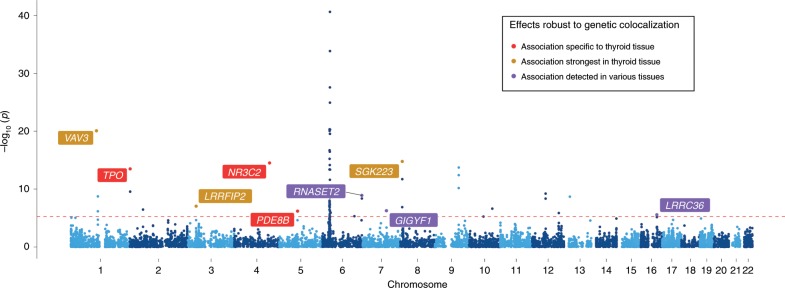
A transcriptome-wide Manhattan plot for genetically predicted gene expression in thyroid tissue and thyroid disease. A Manhattan plot illustrating the association between genetically influenced gene expression derived from thyroid tissue and self-reported thyroid disease in the UK Biobank study. Amongst signals that were robust to genetic colocalization, we identified associations only detected using thyroid tissue (red), associations detected with the strongest evidence in thyroid tissue (i.e., evidence of association in at least two tissues with thyroid being the strongest—yellow), and associations observed across many different tissue types (i.e., evidence of association in at least two tissues where thyroid is not the strongest—purple).

Findings from our extensive analyses can be used to conduct hypothesis-driven investigations of tissue-dependent effects. For example, we hypothesized that genetic variants which influence risk of thyroid disease (defined as self-reported hypothyroidism or myxoedema in the UK Biobank study) may likely act via changes to gene expression in thyroid tissue. Figure 3 illustrates the results of a transcriptome-wide evaluation between thyroid-derived gene expression and thyroid disease using results from our atlas. We identified 68 associations that survived multiple testing (MR *P* < 5.59 × 10^−06^, i.e., 0.05/8946 tests) and 17 of these survived HEIDI filtering (*P* > 0.05; Supplementary Table 3). However, two of these were in the HLA region and should be interpreted with caution due to the extensive LD, which may hinder the reliability of genetic colocalization analyses^22^.

**Fig. 4:**
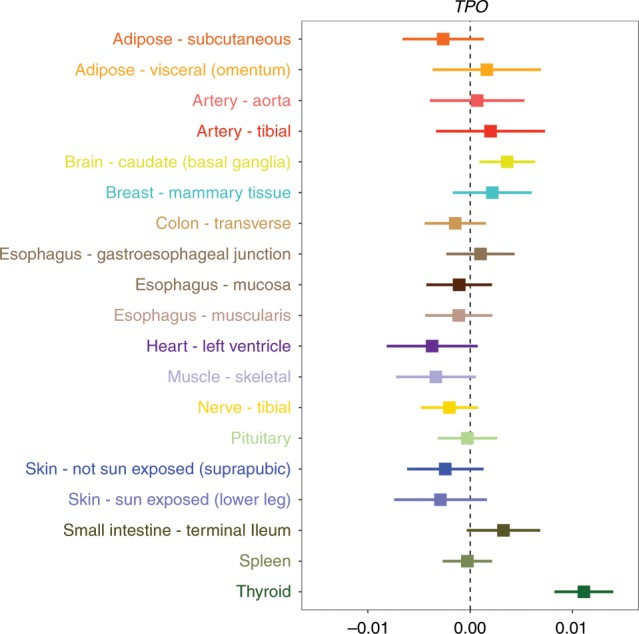
Forest plot illustrating the tissue-dependent association for *TPO* expression and thyroid disease. The horizontal line in this plot indicates the null of beta = 0 and the error bars correspond to 95% confidence intervals. There was only a valid eQTL for 19 of the 48 tissues in GTEx for *TPO*.

We evaluated the association for each of these genetic effects on thyroid disease in all other available tissue types. Although we report these genetic effects based on their corresponding gene symbols, it should be noted that they are based on the MR effect estimates using lead eQTL. We found that in particular two of these associations appeared to be highly tissue specific (*TPO, NR3C2,* and *PDE8B*), as they were only identified in thyroid tissue after correcting for the number of tissues evaluated (Supplementary Tables 4–6). Cross-tissue associations for *TPO* and thyroid disease are illustrated in Fig. 4. These effects provided strong evidence of heterogeneity (Cochran’s *Q* statistic = 104.8, *P* = 7.12 × 10^−14^), which reflects the tissue dependency of associations for *TPO*.

We also identified effects detected most strongly in thyroid tissue, although evidence of association was still identified in other tissue types (*VAV3*, *LRRFIP2,* and *SGK223*, Supplementary Tables 7–9). These results also demonstrate that certain associations appear to be detected across many or all tissue types assessed (e.g., *RNASET2*, Supplementary Table 10). Furthermore, repeating this transcriptome-wide analysis with thyroid disease in all tissue types found that using thyroid-derived gene expression yielded the largest number of associations after excluding results from the HLA region (*n* = 15, Supplementary Table 11).

### Phenome-wide analyses to evaluate tissue-dependent effects

Along with evaluating our results in a transcriptome-wide manner as above, exploring findings in a phenome-wide manner can be a powerful approach to explore pleiotropy. As a demonstration of this, *RPS26* is ubiquitously expressed across all tissue types evaluated by GTEx v7 (Supplementary Fig. 3). Undertaking a phenome-wide scan of this gene’s expression using whole blood suggests that the corresponding variant used as an instrument is highly pleiotropic, as a total of 81 associations survived multiple testing corrections (MR P < 1.27 × 10^−04^ based on 395 traits, Supplementary Data 3, Fig. 5a). *RPS26* therefore appears to be a case in point that genes expressed in many tissues may be more likely to influence multiple different phenotypes.

**Fig. 5:**
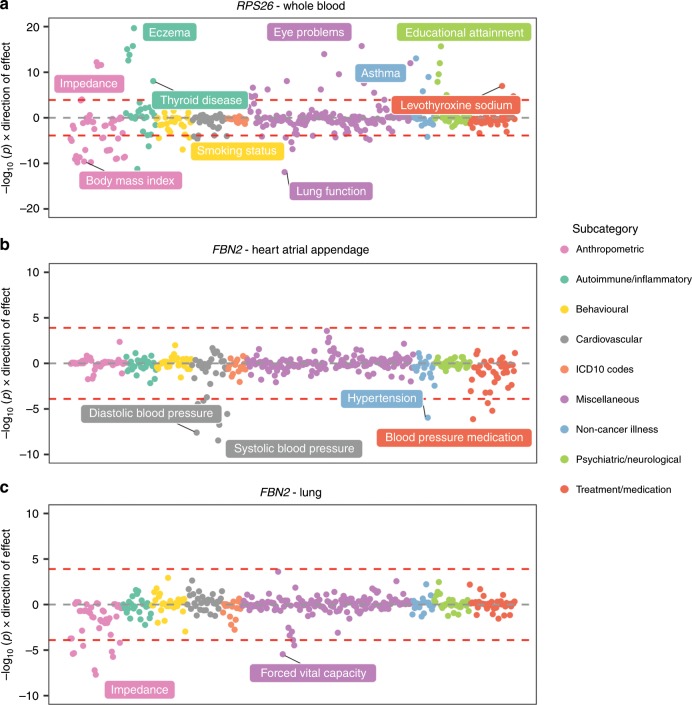
Miami plots illustrating phenome-wide associations between genes in different tissue types. **a** *RPS26* expression derived from whole blood was associated with many diverse traits, **b** *FBN2* expression derived from heart tissue was associated with blood pressure traits, and **c** *FBN2* associations with blood pressure attenuated when analysed using lung-derived data. However, associations with other traits were observed instead.

Investigating phenome-wide associations for genes of interest can also yield insight into tissue-dependent effects. As an example, we evaluated genes in our atlas associated with two traits with a substantial heritable component within the UK Biobank study; diastolic blood pressure (DBP) and forced vital capacity (FVC). We found that *FBN2* expression was linked with both traits in our results, although when using heart tissue-derived data only the effects on blood pressure were observed (Supplementary Data 4, Fig. 5b). However, these associations attenuate when investigating this effect in other tissues types. Moreover, when evaluating phenome-wide associations of *FBN2* using lung tissue-derived eQTL data, we identified evidence of association with FVC (MR *P* = 3.51 × 10^−06^, Supplementary Data 5, Fig. 5c). This is unlikely to be due to differing sample sizes in GTEx given that lung tissue has a larger sample size than heart tissue (*n* = 383 and 264, respectively). Instead findings such as this may be attributed to different eQTL used as IVs for the same gene but within a different tissue type (as is the case for *FBN2*). As such, they may elucidate tissue-dependent regulatory mechanisms that can help explain associations at pleiotropic loci^23^.

### Highlighting unanticipated effects for therapeutic targets

Exploring our associations in a phenome-wide manner may also be valuable for other purposes, such as helping validate whether genes may be viable drug targets^24^. A well-established example of this is the impact of HMG-coenzyme A reductase (HMG-CoA) inhibition using statins, which is known to reduce low-density lipoprotein (LDL) cholesterol levels. However, this is known to also potentially result in increased bodyweight and risk of diabetes^25^.

Undertaking a phenome-wide evaluation of *HMGCR* (the gene responsible for HMG-CoA) using data derived from whole blood supports these findings. We observed strong positive associations between the lead eQTL for this gene and high LDL and total cholesterol levels (Supplementary Data 6, Fig. 6a). There was also evidence of association with lower body mass index (MR *P* = 1.87 × 10^−15^), although the association with self-reported diabetes did not survive phenome-wide corrections (MR *P* = 0.001). Nonetheless, these findings help support the notion that MR analyses can help mimic the findings of randomized control trials^26^ and identify potential on-target side effects of therapeutic intervention^27^. We note however that the tissue analysed may play an important part in such analyses, particularly with respect to the sensitivity of genetic colocalization. Notably, associations with lipid traits using whole blood-derived data did not survive HEIDI corrections, although stronger evidence of colocalization was detected using skeletal muscle tissue (e.g., HEIDI *P* = 0.23 for LDL cholesterol).

**Fig. 6:**
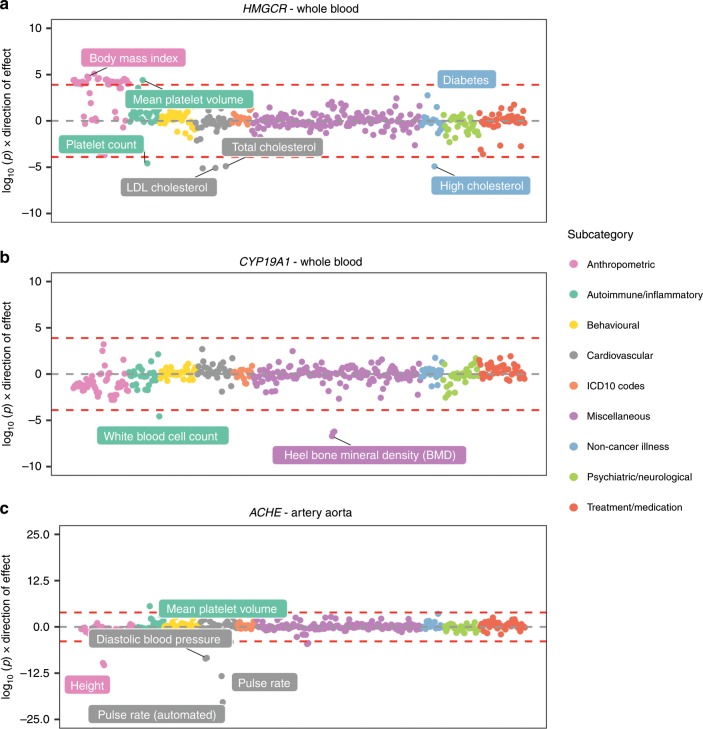
Miami plots representing phenome-wide associations between genes targeted for therapeutic intervention. **a** *HMGCR* associations reflect reported consequences of statins, **b** *CYP19A1* associations support adverse on-target side effects on bone mineral density, and **c** *ACHE* associations demonstrate scope for repurposing opportunities (e.g., possible inhibition to reduce blood pressure).

In terms of targets which are less well established in the literature, our findings highlighted several potential adverse effects by conducting a similar analysis for *CYP19A1* expression using data derived from whole blood (Supplementary Data 7, Fig. 6b). This gene has been previously targeted using the drug Anastrozole to reduce risk of breast cancer^28^, although reported side effects include increased risk of osteoporosis^29^. Our phenome-wide scan of *CYP19A1* provided evidence of this reported on-target adverse effect, as we identified strong evidence of association with heel bone mineral density (BMD; MR *P* = 1.96 × 10^−07^).

Conducting these types of evaluations may also be beneficial for potential drug repositioning opportunities. For instance, *ACHE*, which is a target for drugs used to treat cognitive decline in Alzheimer’s patients, such as galantamine and donepezil^30^. The causal pathway targeted by these drugs would likely be expected to inhibit *ACHE* expression in brain tissue. However, conducting a phenome-wide evaluation for this gene in other tissues (such as artery aorta) indicates that its transcription is associated with higher blood pressure (Supplementary Data 8, Fig. 6c). Further research could therefore explore whether inhibiting this gene’s product may have beneficial implications for hypertension.

### Leveraging findings to prioritize candidate genes

An important challenge in genetic epidemiology is pinpointing the causal gene responsible for association signals detected by GWAS. This is a complex problem for several reasons, including the coexpression that can exist between nearby genes that is often difficult to disentangle^31^. We previously proposed that integrating tissue-specific eQTL data with findings from GWAS may help with such endeavours^9^, along with other properties such as proximity to genes, whether they reside in regulatory regions etc.

For example, rs7500448 is strongly associated with DBP (after adjustment for medication) based on analyses undertaken using data from the UK Biobank study (*P* = 6.3 × 10^−15^, based on linear regression from GWAS). Harnessing all available tissue-dependent results from our atlas allowed us to evaluate associations between nearby genes for which this SNP is an eQTL. Doing so identified only one association signal that survived multiple comparisons, which was *CDH13* using eQTL data derived from the aorta (MR *P* = 2.78 × 10^−08^; Supplementary Data 9, Fig. 7a). This provides strong evidence that *CDH13* may be the causal gene responsible for this effect, and that its expression in the aorta may play a role in blood pressure variation.

**Fig. 7:**
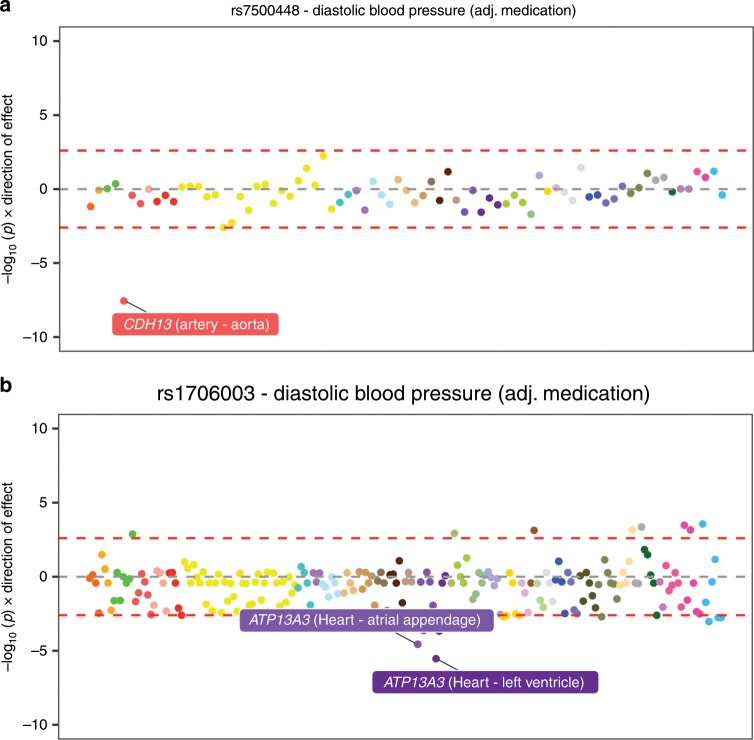
Miami plots illustrating cross-tissue findings at diastolic blood pressure associated loci. Miami plots between all genes whose expression is influenced by proximal SNPs detected by GWAS of diastolic blood pressure (DBP). Points on these plots represent the association between all genes within a 1 Mb distance that these SNPs are eQTL for. Points are coloured in line with those used by GTEx to distinguish tissue types. **a** rs7500448 was strongly associated with DBP based on *CDH13* expression derived from aorta tissue, and **b** rs1706003 was strongly associated with DBP using *ATP13A3* expression data also derived from heart tissue.

This approach may also prove useful in identifying trait-associated variants yet to be discovered by GWAS. For instance, rs1706003 is a SNP associated with blood pressure that may be overlooked based on conventional GWAS corrections (*P* = 1.1 × 10^−07^ with DBP, based on linear regression from GWAS). However, by integrating tissue-specific eQTL data, along with the reduced burden on multiple testing, our analysis provided evidence suggesting that this may be a trait-associated locus yet to be reported by previous studies (Supplementary Data 10, Fig. 7b). The strongest association in this evaluation was with *ATP13A3* expression derived from heart tissue (MR *P* = 3.0 × 10^−06^), which again may help yield mechanistic insight into the causal pathway from genetic variant to phenotype. Furthermore, this is a putative illustration that the nearest gene to a trait-associated SNP is not always the causative one^32^, as the nearest gene to rs1706003 provided weak evidence of association (*TMEM44*, lowest MR *P* across all tissues = 3.1 × 10^−03^). Locus zoom plots for the results highlighted in this section can be found in Supplementary Figs. 4–13.

## Discussion

In this study, we have undertaken a systematic phenome-wide association study to investigate the genetic effects of gene expression across different tissue types. In doing so, we have constructed a putative causal map of tissue-dependent associations across the human transcriptome. We have provided evidence that effects which influence gene expression across multiple tissue types are more likely to be associated with multiple traits. Our results also highlight the value of cross-tissue evaluations in terms of elucidating effects, which depend upon the tissue analysed. We envisage that our findings will facilitate a greater understanding of tissue-specific regulatory mechanisms, which are likely to have translational impact by informing drug target prioritization.

The tissues or cell types which a gene is expressed in is known to reflect the biological processes and functions it carries out^33^. For instance, in this study we demonstrated that the association between *TPO* and thyroid disease appears to be dependent on using expression data derived from thyroid tissue. This gene is responsible for generating thyroid peroxidase and thus plays an important role in regulating thyroid hormones^34^. As such this tissue-specific association reflects the role that this gene has in the thyroid gland. Broadly, we also observed that variants which influence gene expression levels in multiple tissues are more likely to influence multiple complex traits. This suggests that genes expressed in many tissues are more likely to have widespread influence on downstream phenotypic consequences.

In our results, we have demonstrated that phenome-wide evaluations of genes can help elucidate tissue-dependent associations. As an example of this, we show that *FBN2* is associated with various blood pressure traits when using expression data derived from heart tissue. However, when analysing *FBN2* expression using lung-derived data, these effects attenuated, whereas evidence of association with lung function and impedance were detected. This gene is responsible for encoding fibrillin 2, which is a glycoprotein responsible for elastin fibres found in connective tissue^35^. Elastin plays an important role in determining passive mechanical properties of the large arteries and lungs, which helps explain the associations detected in these separate tissues^36,37^. *FBN2* is also associated with other traits and diseases, such as Marfan-like disorder^35^. A better understanding of pleiotropic effects due to regulatory mechanisms may also help to shed light on valid instruments in a conventional MR setting (i.e., between a modifiable environmental risk factor and disease outcome^8^). Specifically, an instrument which is associated with the transcription of a large number of genes (across a diverse range of tissue types) is likely to be more prone to horizontal pleiotropy and therefore invalidate the underlying assumptions of MR.

Phenome-wide evaluations of our findings also have the potential to assist in drug target prioritization. This supports emerging evidence concerning the benefit in using findings from genetic association studies to support therapeutic validation^38,39^. Moreover, this is particularly crucial given the costs of drug development^40^, but also timely given that the highest number of new drugs were approved in 2018^41^. As a proof of concept, we undertook a phenome-wide scan of *HMGCR* which is targeted by statins to reduce elevated cholesterol levels. We identified strong associations with cholesterol traits, as well as findings which reflect reported on-target effects of statins (namely changes in bodyweight and risk of diabetes^25^). So although GWAS datasets typically investigate disease incidence as opposed to disease progression or treatment, evaluations such as these may still be useful for therapeutic validation^24^. We also note that the ideal tissue type for a specific hypothesis may not always be feasible based on current datasets (e.g., liver tissue to investigate the association between *HMGCR* expression with LDL cholesterol). Moreover, the ideal tissue (or tissues with regards to analysing joint effects) may not always be well documented in the literature.

Our results can also be used to flag on-target effects which are less well established in pharmacogenetics. For instance, our evaluation of *CYP19A1* suggested that inhibiting this target may result in lower BMD. This finding supports a side effect previously reported for the anticancer drug anastrozole which targets this gene^29^. The therapeutic benefit of statins on lower risk of coronary heart disease has been found to outweigh the adverse side effects on diabetes risk^42^. Uncovering potential side effects for other drug targets should motivate future endeavours to evaluate whether the benefits of therapeutic intervention outweigh the possible drawbacks. Similar evaluations may also help highlight the potential drug repurposing and repositioning opportunities. We provide an example of this suggesting that targeting *ACHE* (originally targeted to treat cognitive decline in Alzheimer’s patients) may help lower blood pressure levels. There are likely many other potential associations from our analyses which may highlight the potential drug repurposing/repositioning opportunities.

In the final series of analyses in our study, we propose that integrating tissue-specific eQTL data into GWAS analyses may help highlight genes responsible for association signals. Our approach therefore supports the notion of triangulation in epidemiology, whereby many lines of evidence are needed to support robust conclusions (i.e., colocalization of eQTL and GWAS effects)^43^. The examples we have showcased in this regard involve SNPs associated with blood pressure, where we prioritize *CDH13* and *ATP13A3* as genes which may be responsible for these effects. *CDH13* is a regulator of vascular wall remodelling and angiogenesis^44^, and *ATP13A3* has recently been implicated in pulmonary arterial hypertension susceptibility through rare loss of function analyses^45,46^. However, although there are likely many instances where integrating tissue-specific eQTL data can help pinpoint genes responsible for GWAS associations, this may not always be possible due to the complexities of coexpression and widely expressed genes^47^. Moreover, we emphasize that integrating gene expression data to help highlight potential genes underlying GWAS hits should only be considered as evidence of prioritizing likely candidates which functional analyses can investigate in detail.

Endeavours which continue to generate increasingly large-scale tissue-specific molecular datasets will facilitate data mining opportunities across the human transcriptome^48^. Although the current sample sizes have meant that the analyses in this study have been restricted to using lead eQTLs only, future efforts will benefit from leveraging multiple valid instruments within a MR framework. This will also facilitate the application of various sensitivity analyses that can be undertaken for MR analyses, such as leave-one out analyses and the MR-Egger approach^49^. Nonetheless, techniques in genetic colocalization will likely continue to play an important role in discerning whether associations are detected due to shared causal variants. We also note that the inference of colocalization methods may be limited when evaluating associations at loci of dense LD (such as the HLA region of the genome).

Furthermore, the approach used in our study (as with all alternatives to date) is unable to robustly rule out that findings may be influenced by molecular horizontal pleiotropy. This is the process whereby a genetic variant influences gene expression and a complex trait via two independent biological pathways. It may also be possible that a variant may influence complex trait variation via a posttranscriptional mechanism, which should be worthwhile evaluating in future studies once tissue-specific protein QTL data becomes accessible in large sample sizes. Moreover, we note that cross-tissue inference of our findings has the caveat of differing sample sizes in GTEx for different tissues. It is therefore important to take into account the sample size for each tissue type when interpreting findings, as this has an influence on the power to detect lead eQTLs for genes (Supplementary Table 12). We also note that additional consideration should be taken with regard to any covariates that were adjusted for in the original GWAS for an outcome of interest (e.g., the adjustment for medication for measures of blood pressure).

When evaluating associations in our results, it is important to remember that they are based on SNP effect sizes which are often relatively modest^50^, but potentially effective throughout the life course. Therefore, when evaluating our results for the purpose of drug validation, it is worth noting that pharmaceutical targeting of a protein is likely to have a larger effect on protein levels, but over a shorter time period. We found that the results from this study regarding possible drug targets (i.e., *HMGCR, CYP19A1*, and *ACHE*) were comparable to those detected using a transcriptome-wide association study (TWAS) by querying findings from the TWAS hub^51^. However, other findings such as the association between *TPO* and thyroid disease were not identified using this alternative method. We therefore propose although the analysis used in this study is comparable to that of TWAS, both approaches may prove useful in detecting evidence of association which the other may overlook.

Furthermore, we note that using an alternative colocalization method to the HEIDI test, such as coloc^52^, enloc^53^, or eCAVIAR^11^, in our analyses may have detected evidence for different association signals. Whilst the HEIDI method is not prone to some of the caveats of these approaches (such as sensitivity to prior distributions), it may not always be capable of detecting heterogeneity under certain circumstances. Finally, evidence from the literature suggests that the expression for an increasingly large proportion of protein-coding genes is influenced by multiple independent variants^54^. Future study designs should therefore take this into account as tissue-specific datasets increase in scale (Supplementary Note 1). Such endeavours may also wish to use an updated reference panel rather than the 1000 genomes data used in this study to improve LD estimation between SNPs.

The results we have highlighted in our study are likely just the tip of the iceberg in terms of findings from our atlas that provide insight into the regulatory mechanisms underlying human complex traits. Although studies have used GTEx data to investigate tissue specificity previously, their results are not easily accessible in a format that allow transcriptome-wide, phenome-wide, or cross-tissue evaluations. Our web application should prove fruitful for users in this regard, facilitating in-depth evaluations of current findings or motivating innovative research hypotheses. Future endeavours which harness increasingly large-scale molecular datasets derived from different tissue types will enhance our capability to understand the determinants of complex disease.

## Methods

### Data resources

Tissue-specific eQTL data was obtained from the GTEx project (v7; https://gtexportal.org/home/). Only 48 of the 53 tissues available from GTEx v7 were analysed as each of the remaining 5 had fewer than 50 samples. As anticipated, there was a strong positive correlation between the number of unique genes eligible for analysis in a single tissue type compared with the sample size of that tissue (Supplementary Table 13). We also obtained eQTL data derived from whole blood in 31,684 individuals made available by the eQTLGen consortium (http://www.eqtlgen.org). GWAS summary statistics were obtained from the Neale Lab analyses of UK Biobank data and consortia who have made their results publicly available (a full list can be found in Supplementary Data 1)^55–71^. All ethical approvals for these analyses can be located in the corresponding studies.

### Statistical analyses

We conducted analyses using the summary-data-based MR (SMR) method (v0.710). A reference panel of European individuals from the 1000 genomes project (phase 3) was used to compute LD estimation for all analyses^72^. As proposed previously^73^, only *cis*-eQTL were used as IVs (based on <1 Mb of associated probe). This is to reduce the likelihood of associations attributed to horizontal pleiotropy to which *trans*-effects are more prone. The summary statistics from GWAS analysed in our study are typically restricted to disease incidence as opposed to disease progression. Furthermore, these effect estimates do not involve analysis of repeated measures.

Consequently, only lead eQTLs for each gene were used as IVs given that very few genes could be robustly instrumented with multiple independent SNPs in the GTEx dataset. In the few instances where genes from GTEx could be instrumented using multiple independent instruments (based on *r*^2^ < 0.001), only the lead eQTL based on observed *P* values was used as an IV. This approach was also applied when analysing data from the eQTLGen consortium despite the larger sample sizes, for consistency when comparing associations between dataset. We defined eQTL based on a lenient *P* value threshold of *P* < 1 × 10^−04^, maximizing the number of possible genes analysed across tissues but also allowing readers to filter out associations should they wish to apply a more stringent threshold. To assess instrument strength based on this lenient threshold we calculated *F*-statistics as proposed by Bowden et al.^74^:

$$F_j = \frac{{\gamma _j^2}}{{\sigma _{Xj}^2}}$$

where *γ*_*j*_ is the SNP-exposure association and *σ*_*Xj*_ is the standard deviation for the SNP-exposure association for variant *j*.

An analysis of variance model was applied to investigate the association between the number of traits and number of tissue types detected for all lead eQTL in our curated results (i.e., *P* < 5 × 10^−08^ that were also robust to a strict HEIDI correction of *P* > 0.05). A strict lead eQTL threshold of *P* < 5 × 10^−08^ was also applied to assemble this curated set of results. However, it is also possible that genomic properties (such as LD structure, proximity to nearest gene etc) may influence the number of traits which multitissue eQTLs are associated with. Therefore, we adjusted our analysis for minor allele frequencies, LD score, and distance to gene expression probe for lead eQTL. Furthermore, associations detected using eQTLGen whole blood-derived data were removed from this analysis to reduce any bias which may be attributed to the large sample size of this dataset. Pearson’s *r*^2^ was calculated to compare the correlation between the number of associations identified with the number of tissues they were detected across for each eQTL clustering by their associated gene.

By default, our web application displays multiple testing comparisons based on Bonferroni correction for the number of tests undertaken in the search query. Subsequently, HEIDI corrections are applied based on the number of associations which survived multiple testing in this look up^20,75^. All analyses were undertaken using R (version 3.5.1). The R package ‘shiny’ v1.1 was used to develop the web application. The R packages ‘manhattanly’ v0.2 and ‘highcharter’ v0.5 were used to generate interactive plots. Figures in this manuscript were generated using ‘ggplot2’ v2.2.1.

### Reporting summary

Further information on research design is available in the Nature Research Reporting Summary linked to this article.

## Supplementary information

Dataset 1

Dataset 2

Dataset 3

Dataset 4

Dataset 5

Dataset 6

Dataset 7

Dataset 8

Dataset 9

Dataset 10

Dataset 11

Supplementary Information

Reporting Summary

## Data Availability

All results from the analyses undertaken in this study can be downloaded using our web application (http://mrcieu.mrsoftware.org/Tissue_MR_atlas/).

## Code availability

The code used in this study can be found at the SMR homepage (https://cnsgenomics.com/software/smr/).
