# Supplementary material for: A transcriptome-wide Mendelian randomization study to uncover tissue-dependent regulatory mechanisms across the human phenome
Source: Nat Commun. 2020 Jan 10;11:185. doi: 10.1038/s41467-019-13921-9 (PMC6954187; doi:10.1038/s41467-019-13921-9)
Supplement: Supplementary file 12 — Supplementary Information [file 41467_2019_13921_MOESM12_ESM.docx]

**Supplementary Material:**

**Tables:**

**Supplementary Table 1: A list of the tissue types evaluated in this study using expression quantitative trait loci data**

*A list of tissues types evaluated in this study and their characteristics.
All data was obtained from the GTEx consortium (https://gtexportal.org/home/) and the eQTLGen project (http://www.eqtlgen.org/)*

| **Tissue type** | **Sample size** | **Resource** |
| --- | --- | --- |
| Adipose - Subcutaneous | 385 | GTEx consortium |
| Adipose - Visceral (Omentum) | 313 | GTEx consortium |
| Adrenal Gland | 175 | GTEx consortium |
| Artery - Aorta | 267 | GTEx consortium |
| Artery - Coronary | 152 | GTEx consortium |
| Artery - Tibial | 388 | GTEx consortium |
| Brain - Amygdala | 88 | GTEx consortium |
| Brain - Anterior cingulate cortex (BA24) | 109 | GTEx consortium |
| Brain - Caudate (basal ganglia) | 144 | GTEx consortium |
| Brain - Cerebellar Hemisphere | 125 | GTEx consortium |
| Brain - Cerebellum | 154 | GTEx consortium |
| Brain - Cortex | 136 | GTEx consortium |
| Brain - Frontal Cortex (BA9) | 118 | GTEx consortium |
| Brain - Hippocampus | 111 | GTEx consortium |
| Brain - Hypothalamus | 108 | GTEx consortium |
| Brain - Nucleus accumbens (basal ganglia) | 130 | GTEx consortium |
| Brain - Putamen (basal ganglia) | 111 | GTEx consortium |
| Brain - Spinal cord (cervical c-1) | 83 | GTEx consortium |
| Brain - Substantia nigra | 80 | GTEx consortium |
| Breast Mammary Tissue | 251 | GTEx consortium |
| Cells - EBV-transformed lymphocytes | 117 | GTEx consortium |
| Cells - Transformed fibroblasts | 300 | GTEx consortium |
| Colon - Sigmoid | 203 | GTEx consortium |
| Colon - Transverse | 246 | GTEx consortium |
| Esophagus - Gastroesophageal Junction | 213 | GTEx consortium |
| Esophagus - Mucosa | 358 | GTEx consortium |
| Esophagus - Muscularis | 335 | GTEx consortium |
| Heart - Atrial Appendage | 264 | GTEx consortium |
| Heart - Left Ventricle | 272 | GTEx consortium |
| Liver | 153 | GTEx consortium |
| Lung | 383 | GTEx consortium |
| Minor Salivary Gland | 85 | GTEx consortium |
| Muscle - Skeletal | 491 | GTEx consortium |
| Nerve - Tibial | 361 | GTEx consortium |
| Ovary | 122 | GTEx consortium |
| Pancreas | 220 | GTEx consortium |
| Pituitary | 157 | GTEx consortium |
| Prostate | 132 | GTEx consortium |
| Skin - Not Sun Exposed (Suprapubic) | 335 | GTEx consortium |
| Skin - Sun Exposed (Lower leg) | 414 | GTEx consortium |
| Small Intestine - Terminal Ileum | 122 | GTEx consortium |
| Spleen | 146 | GTEx consortium |
| Stomach | 237 | GTEx consortium |
| Testis | 225 | GTEx consortium |
| Thyroid | 399 | GTEx consortium |
| Uterus | 101 | GTEx consortium |
| Vagina | 106 | GTEx consortium |
| Whole Blood | 369 | GTEx consortium |
| Whole Blood | 31,684 | eQTLGen |

**Supplementary Table 2: Groups of tissue types evaluated**

A list of tissues types evaluated in this analysis and their allocated group

All data was obtained from the GTEx consortium (https://gtexportal.org/home/) and the eQTLGen project (http://www.eqtlgen.org/)

| **Tissue type** | **Group** |
| --- | --- |
| Adipose - Subcutaneous | Adipose |
| Adipose - Visceral (Omentum) | Adipose |
| Adrenal Gland | Adrenal Gland |
| Artery - Aorta | Artery |
| Artery - Coronary | Artery |
| Artery - Tibial | Artery |
| Brain - Amygdala | Brain |
| Brain - Anterior cingulate cortex (BA24) | Brain |
| Brain - Caudate (basal ganglia) | Brain |
| Brain - Cerebellar Hemisphere | Brain |
| Brain - Cerebellum | Brain |
| Brain - Cortex | Brain |
| Brain - Frontal Cortex (BA9) | Brain |
| Brain - Hippocampus | Brain |
| Brain - Hypothalamus | Brain |
| Brain - Nucleus accumbens (basal ganglia) | Brain |
| Brain - Putamen (basal ganglia) | Brain |
| Brain - Spinal cord (cervical c-1) | Brain |
| Brain - Substantia nigra | Brain |
| Breast Mammary Tissue | Breast Mammary Tissue |
| Cells - EBV-transformed lymphocytes | Cells |
| Cells - Transformed fibroblasts | Cells |
| Colon - Sigmoid | Colon |
| Colon - Transverse | Colon |
| Esophagus - Gastroesophageal Junction | Esophagus |
| Esophagus - Mucosa | Esophagus |
| Esophagus - Muscularis | Esophagus |
| Heart - Atrial Appendage | Heart |
| Heart - Left Ventricle | Heart |
| Liver | Liver |
| Lung | Lung |
| Minor Salivary Gland | Minor Salivary Gland |
| Muscle - Skeletal | Muscle |
| Nerve - Tibial | Nerve - Tibial |
| Ovary | Ovary |
| Pancreas | Pancreas |
| Pituitary | Pituitary |
| Prostate | Prostate |
| Skin - Not Sun Exposed (Suprapubic) | Skin |
| Skin - Sun Exposed (Lower leg) | Skin |
| Small Intestine - Terminal Ileum | Small Intestine |
| Spleen | Spleen |
| Stomach | Stomach |
| Testis | Testis |
| Thyroid | Thyroid |
| Uterus | Uterus |
| Vagina | Vagina |
| Whole Blood | Whole Blood |
| Whole Blood | Not assessed in this analysis |

**Supplementary Table 3: A genome-wide evaluation of thyroid disease using thyroid-derived gene expression data**

*Genome-wide associations between genes expressed in thyroid tissue and thyroid disease in the UK Biobank (defined as self-reported hypothyroidism or myxoedema)*

| **Gene** | **CHR** | **BP** | **Lead SNP** | **Beta** | **SE** | **P** | **P_HEIDI** | **nSNP_HEIDI** |
| --- | --- | --- | --- | --- | --- | --- | --- | --- |
| *HLA-DPB2* | 6 | 33091335 | rs1811359 | -0.00977 | 0.001039 | 5.17E-21 | 0.480846 | 20 |
| *SGK223* | 8 | 8209633 | rs2921053 | 0.007234 | 0.000909 | 1.68E-15 | 0.6367311 | 20 |
| *NR3C2* | 4 | 149182881 | rs6822359 | -0.0087 | 0.001103 | 3.09E-15 | 0.1468486 | 20 |
| *TPO* | 2 | 1462739 | rs11675342 | 0.011123 | 0.001466 | 3.27E-14 | 0.4305674 | 20 |
| *CTA-398F10.2* | 8 | 8316633 | rs2921053 | 0.008887 | 0.001263 | 1.96E-12 | 0.0537659 | 20 |
| *AC144450.1* | 2 | 1587161 | rs11675342 | 0.015788 | 0.002502 | 2.78E-10 | 0.0575375 | 20 |
| *RNASET2* | 6 | 167356832 | rs1811120 | -0.02165 | 0.003562 | 1.22E-09 | 0.0566755 | 20 |
| *HCP5* | 6 | 31406881 | rs3094012 | -0.02094 | 0.003697 | 1.48E-08 | 0.0973466 | 20 |
| *LRRFIP2* | 3 | 37156070 | rs6786584 | -0.00326 | 0.000609 | 9.01E-08 | 0.7986434 | 20 |
| *NKX2-3* | 10 | 101294484 | rs10748781 | 0.007116 | 0.001378 | 2.42E-07 | 0.3433124 | 20 |
| *RP11-554J4.1* | 2 | 55845157 | rs6736996 | 0.004407 | 0.000865 | 3.51E-07 | 0.0504041 | 20 |
| *GIGYF1* | 7 | 100282100 | rs221800 | -0.0124 | 0.002477 | 5.48E-07 | 0.1895816 | 20 |
| *PDE8B* | 5 | 76615953 | rs6885099 | 0.005102 | 0.001025 | 6.5E-07 | 0.0528405 | 20 |
| *RP3-340B19.3* | 6 | 35512927 | rs1984117 | -0.00654 | 0.001347 | 1.22E-06 | 0.1144523 | 20 |
| *LRRC36* | 16 | 67389903 | rs2059237 | 0.007171 | 0.001529 | 2.74E-06 | 0.1720311 | 20 |
| *AC009061.1* | 16 | 67467763 | rs13333582 | 0.010653 | 0.002273 | 2.77E-06 | 0.5671552 | 20 |
| *HSD11B2* | 16 | 67468005 | rs3893749 | 0.010249 | 0.002207 | 3.42E-06 | 0.7817214 | 20 |

| **Supplementary Table 4: A cross-tissue evaluation of the association between *TPO* expression and**  **thyroid disease** |  |
| --- | --- |
| *Associations between TPO expressed in various tissues and thyroid disease in the UK Biobank (defined as self-reported hypothyroidism or myxoedema)* | |

| **Tissue** | **Lead eQTL** | **MR Beta** | **MR SE** | **MR P** | **HEIDI P** | **HEIDI nSNPs** |
| --- | --- | --- | --- | --- | --- | --- |
| Thyroid | rs11675342 | 0.0111228 | 0.00146598 | 3.27E-14 | 0.4305674 | 20 |
| Brain - Caudate (basal ganglia) | rs79851893 | 0.00362583 | 0.00139688 | 0.00944055 | 0.362353 | 4 |
| Small Intestine - Terminal Ileum | rs6717701 | 0.00325691 | 0.001834 | 0.07575685 | 0.3015487 | 12 |
| Muscle - Skeletal | rs28591627 | -0.00334736 | 0.00199369 | 0.09315602 | 0.05775106 | 20 |
| Heart - Left Ventricle | rs28591627 | -0.00371759 | 0.00227167 | 0.1017357 | 0.06135933 | 20 |
| Nerve - Tibial | rs4927612 | -0.00201859 | 0.00142831 | 0.1575776 | 0.2788568 | 20 |
| Adipose - Subcutaneous | rs2175977 | -0.00263781 | 0.00203312 | 0.1944879 | 0.1368324 | 20 |
| Skin - Not Sun Exposed (Suprapubic) | rs2175977 | -0.002432 | 0.00190487 | 0.2016974 | 0.6532443 | 13 |
| Skin - Sun Exposed (Lower leg) | rs6588678 | -0.00290313 | 0.00231402 | 0.2096295 | 0.4966345 | 13 |
| Breast - Mammary Tissue | rs72766713 | 0.00216366 | 0.00196942 | 0.2719304 | 0.401314 | 20 |
| Colon - Transverse | rs6588671 | -0.00145614 | 0.00152797 | 0.3405951 | 0.295882 | 20 |
| Artery - Tibial | rs28776240 | 0.00199157 | 0.00272119 | 0.4642448 | 0.2718872 | 14 |
| Esophagus - Muscularis | rs3772074 | -0.00111596 | 0.00167827 | 0.5060861 | 0.05996947 | 20 |
| Esophagus - Mucosa | rs938330 | -0.00107395 | 0.00163406 | 0.5110345 | 0.05306241 | 20 |
| Adipose - Visceral (Omentum) | rs13430002 | 0.0016291 | 0.00270808 | 0.5474596 | 0.4997095 | 20 |
| Esophagus - Gastroesophageal Junction | rs6711500 | 0.00100621 | 0.00170166 | 0.5543138 | 0.01064589 | 15 |
| Artery - Aorta | rs79410013 | 0.000685586 | 0.00235863 | 0.7713027 | NA | NA |
| Spleen | rs12473881 | -0.00027029 | 0.00123458 | 0.8267051 | 0.6285268 | 7 |
| Pituitary | rs3755552 | -0.00026265 | 0.00148273 | 0.8593982 | 0.05409084 | 10 |

| **Supplementary Table 5: A cross-tissue evaluation of the association between *NR3C2* expression and**  **thyroid disease** |
| --- |
| *Associations between NR3C2 expressed in various tissues and thyroid disease in the UK Biobank (defined as self-reported hypothyroidism or myxoedema)* |

| **Tissue** | **Lead eQTL** | **MR Beta** | **MR SE** | **MR P** | **HEIDI P** | **HEIDI nSNPs** |
| --- | --- | --- | --- | --- | --- | --- |
| Thyroid | rs6822359 | -0.00870131 | 0.00110319 | 3.09E-15 | 0.1468486 | 20 |
| Vagina | rs115645143 | 0.00396528 | 0.00271081 | 0.1435324 | NA | NA |
| Artery - Tibial | rs34927488 | 0.00630779 | 0.004502 | 0.1611811 | NA | NA |
| Brain - Hippocampus | rs61762827 | 0.00141497 | 0.00111146 | 0.2029902 | NA | NA |
| Lung | rs79077421 | 0.0032911 | 0.00347289 | 0.3433049 | NA | NA |
| Whole Blood | rs143081997 | -0.000503305 | 0.00435103 | 0.9079102 | NA | NA |

| **Supplementary Table 6: A cross-tissue evaluation of the association between *PDE8B* expression and**  **thyroid disease** |
| --- |
| *Associations between PDE8B expressed in various tissues and thyroid disease in the UK Biobank (defined as self-reported hypothyroidism or myxoedema)* |

| **Tissue** | **Lead eQTL** | **MR Beta** | **MR SE** | **MR P** | **HEIDI P** | **HEIDI nSNPs** |
| --- | --- | --- | --- | --- | --- | --- |
| Thyroid | rs6885099 | 0.00510153 | 0.00102528 | 6.50E-07 | 0.05284052 | 20 |
| Adrenal Gland | rs7707719 | -0.0073618 | 0.00204884 | 0.0003267 | 0.0133 | 13 |
| Lung | rs2928167 | -0.0047971 | 0.00169323 | 0.0046102 | 0.05012191 | 13 |
| Cells - Transformed fibroblasts | rs2560076 | 0.0018531 | 0.00072693 | 0.01079687 | 0.4805996 | 20 |
| Esophagus - Muscularis | rs6858923 | 0.00511821 | 0.0021038 | 0.01498073 | 0.7364043 | 20 |
| Brain - Cerebellum | rs6453332 | 0.00315637 | 0.00135396 | 0.01974218 | 0.01241199 | 20 |
| Whole Blood | rs335630 | 0.00313707 | 0.00134744 | 0.01990344 | 0.6001912 | 20 |
| Brain - Cerebellar Hemisphere | rs335630 | 0.00295649 | 0.00129432 | 0.02235994 | 0.2923763 | 20 |
| Prostate | rs7707014 | -0.0052355 | 0.00242062 | 0.03055169 | 0.5954801 | 4 |
| Muscle - Skeletal | rs10805911 | 0.00239541 | 0.00115748 | 0.03849771 | 0.2698552 | 20 |
| Whole Blood (eQTLGen) | rs163036 | 0.00359128 | 0.00190862 | 0.05988886 | 0.3047567 | 20 |
| Testis | rs79008179 | 0.00338555 | 0.00182059 | 0.06294357 | 0.5466096 | 10 |
| Spleen | rs922538 | 0.00182707 | 0.00102126 | 0.07360841 | 0.4311411 | 20 |
| Liver | rs34802194 | -0.0012948 | 0.00077844 | 0.09625311 | 0.0071983 | 20 |
| Brain - Spinal cord (cervical c-1) | rs7732130 | -0.0017711 | 0.0011022 | 0.1080746 | 0.03345608 | 4 |
| Esophagus - Mucosa | rs335632 | 0.00142571 | 0.00098423 | 0.1474641 | 0.1259946 | 20 |
| Nerve - Tibial | rs335632 | 0.00169522 | 0.00117281 | 0.148338 | 0.2163936 | 20 |
| Skin - Sun Exposed (Lower leg) | rs335632 | 0.00210864 | 0.00146509 | 0.150078 | 0.05828521 | 20 |
| Artery - Aorta | rs10045543 | 0.00343223 | 0.00244981 | 0.161208 | 0.242594 | 18 |
| Stomach | rs10063453 | 0.00426751 | 0.00342294 | 0.2124934 | 0.2433025 | 4 |
| Pancreas | rs6453294 | 0.00105723 | 0.00088739 | 0.2335 | 0.3123715 | 20 |
| Cells - EBV-transformed lymphocytes | rs335660 | 0.00069319 | 0.00060943 | 0.2553528 | 0.1148298 | 20 |
| Brain - Putamen (basal ganglia) | rs72773778 | 0.00113445 | 0.00099913 | 0.2561916 | NA | NA |
| Small Intestine - Terminal Ileum | rs335660 | 0.00217921 | 0.00196306 | 0.2669506 | 0.9530127 | 3 |
| Esophagus - Gastroesophageal Junction | rs4259151 | 0.00255628 | 0.00260834 | 0.3270656 | 0.1131653 | 3 |
| Colon - Sigmoid | rs11750710 | -0.000749 | 0.00090666 | 0.4087585 | 0.03589828 | 20 |
| Brain - Frontal Cortex (BA9) | rs3846675 | 0.00133119 | 0.00172782 | 0.4410381 | 0.1006292 | 12 |
| Skin - Not Sun Exposed (Suprapubic) | rs10474515 | 0.00085859 | 0.00114033 | 0.4514932 | 0.0628826 | 20 |
| Colon - Transverse | rs11741458 | -0.0009908 | 0.00205728 | 0.6300857 | 0.01804601 | 20 |
| Adipose - Subcutaneous | rs6864250 | 0.00038157 | 0.00375499 | 0.91906 | 0.3097405 | 8 |

| **Supplementary Table 7: A cross-tissue evaluation of the association between *VAV3* expression and**  **thyroid disease** |  |
| --- | --- |
| *Associations between VAV3 expressed in various tissues and thyroid disease in the UK Biobank (defined as self-reported hypothyroidism or myxoedema)* | |

| **Tissue** | **Lead eQTL** | **MR Beta** | **MR SE** | **MR P** | **HEIDI P** | **HEIDI nSNPs** |
| --- | --- | --- | --- | --- | --- | --- |
| Thyroid | rs78495697 | 0.00958243 | 0.00102466 | 8.61E-21 | 0.0170308 | 20 |
| Lung | rs17020055 | 0.0147307 | 0.00230961 | 1.79E-10 | 0.0055322 | 20 |
| Whole Blood | rs12566129 | 0.0267679 | 0.00467955 | 1.06E-08 | 0.0021437 | 20 |
| Nerve - Tibial | rs12566129 | 0.0158675 | 0.0029672 | 8.91E-08 | 1.02E-05 | 20 |
| Heart - Atrial Appendage | rs4915080 | 0.0168313 | 0.00316959 | 1.09E-07 | 0.0062493 | 20 |
| Adipose - Subcutaneous | rs12566129 | 0.0173144 | 0.00352042 | 8.73E-07 | 0.0104795 | 15 |
| Adrenal Gland | rs79748332 | 0.0104683 | 0.00220846 | 2.14E-06 | 0.0171365 | 9 |
| Spleen | rs79748332 | 0.0126031 | 0.00277616 | 5.63E-06 | 0.0084083 | 8 |
| Esophagus - Muscularis | rs2169077 | 0.0205989 | 0.0046746 | 1.05E-05 | 0.2338029 | 8 |
| Muscle - Skeletal | rs79748332 | 0.0219714 | 0.00547915 | 6.07E-05 | 0.0383141 | 3 |
| Brain - Cerebellum | rs72705622 | 0.0187896 | 0.00520046 | 0.000303 | 0.0304203 | 6 |
| Esophagus - Mucosa | rs4384261 | 0.0144422 | 0.00534844 | 0.006928 | 0.3696656 | 3 |
| Skin - Sun Exposed (Lower leg) | rs610219 | -0.0103594 | 0.00418113 | 0.013224 | 0.3840464 | 5 |
| Adipose - Visceral (Omentum) | rs380677 | -0.002769 | 0.00181103 | 0.126273 | 0.3274763 | 8 |
| Heart - Left Ventricle | rs12409796 | -0.00195982 | 0.00144695 | 0.175594 | 0.3762198 | 20 |
| Brain - Hippocampus | rs4626916 | 0.00177614 | 0.00138643 | 0.200164 | 0.0162657 | 8 |
| Liver | rs1410403 | -0.00292466 | 0.00252403 | 0.246568 | 0.8323544 | 5 |
| Pituitary | rs1219765 | -0.00137234 | 0.00186844 | 0.462655 | NA | NA |
| Pancreas | rs149648027 | -0.00120678 | 0.00199718 | 0.545682 | NA | NA |
| Cells - Transformed fibroblasts | rs189561381 | 0.000715959 | 0.00188294 | 0.703771 | NA | NA |

| **Supplementary Table 8: A cross-tissue evaluation of the association between *LRRFIP2* expression**  **and thyroid disease** |  |  |  |  |  |  |
| --- | --- | --- | --- | --- | --- | --- |
| *Associations between LRRFIP2 expressed in various tissues and thyroid disease in the UK Biobank (defined as self-reported hypothyroidism or*  *myxoedema)* | | | | | | |

| **Tissue** | **Lead eQTL** | **MR Beta** | **MR SE** | **MR P** | **HEIDI P** | **HEIDI nSNPs** |
| --- | --- | --- | --- | --- | --- | --- |
| Thyroid | rs6786584 | -0.00325712 | 0.000609301 | 9.01E-08 | 0.7986434 | 20 |
| Muscle - Skeletal | rs7638620 | -0.0076608 | 0.00155062 | 7.79E-07 | 0.402716 | 20 |
| Artery - Aorta | rs6772548 | -0.00705462 | 0.00152546 | 3.75E-06 | 0.9048771 | 20 |
| Esophagus - Muscularis | rs9854956 | -0.00927518 | 0.00201366 | 4.10E-06 | 0.6247396 | 20 |
| Skin - Sun Exposed (Lower leg) | rs55898028 | 0.00901099 | 0.00198402 | 5.58E-06 | 0.1175134 | 20 |
| Cells - Transformed fibroblasts | rs7628085 | -0.0111714 | 0.00256185 | 1.30E-05 | 0.3311283 | 20 |
| Skin - Not Sun Exposed (Suprapubic) | rs9810355 | 0.0107981 | 0.00249283 | 1.48E-05 | 0.1862264 | 20 |
| Whole Blood | rs9831178 | 0.018157 | 0.00432142 | 2.65E-05 | 0.4647984 | 20 |
| Nerve - Tibial | rs9839105 | -0.00849671 | 0.00203082 | 2.87E-05 | 0.3425439 | 20 |
| Esophagus - Gastroesophageal Junction | rs4678920 | -0.00937071 | 0.00226213 | 3.44E-05 | 0.7105233 | 20 |
| Testis | rs1558527 | -0.0145848 | 0.003538 | 3.75E-05 | 0.9427672 | 20 |
| Artery - Tibial | rs10849 | -0.0102666 | 0.00263464 | 9.75E-05 | 0.05466592 | 20 |
| Brain - Hippocampus | rs4678943 | -0.00862658 | 0.00226899 | 0.000143565 | 0.3375603 | 20 |
| Brain - Amygdala | rs4431051 | -0.00585369 | 0.00154586 | 0.000152677 | 0.8445884 | 20 |
| Brain - Caudate (basal ganglia) | rs4431051 | -0.00912969 | 0.00249145 | 0.000247914 | 0.626086 | 18 |
| Brain - Cortex | rs4678561 | -0.0098553 | 0.00282165 | 0.000478077 | 0.7407739 | 20 |
| Brain - Substantia nigra | rs113479434 | -0.009175 | 0.00267546 | 0.000605086 | 0.2415211 | 9 |
| Brain - Cerebellum | rs6550458 | -0.0140551 | 0.00411683 | 0.000639998 | 0.7948831 | 10 |
| Colon - Sigmoid | rs9876275 | -0.0110519 | 0.00334273 | 0.000945548 | 0.7666082 | 14 |
| Brain - Putamen (basal ganglia) | rs11714716 | -0.0072525 | 0.00219553 | 0.000955514 | 0.3366896 | 19 |
| Esophagus - Mucosa | rs4678938 | 0.0282324 | 0.00872274 | 0.001209456 | 0.9679637 | 11 |
| Adrenal Gland | rs11706926 | -0.00800502 | 0.002568 | 0.001825672 | 0.2661732 | 11 |
| Heart - Atrial Appendage | rs9839933 | 0.0168567 | 0.00552669 | 0.002288075 | 0.3079751 | 10 |
| Pancreas | rs2276808 | -0.0119237 | 0.00397214 | 0.002683634 | 0.2313904 | 12 |
| Liver | rs374951 | -0.0055673 | 0.00191027 | 0.003563721 | 0.7663545 | 6 |
| Prostate | rs73068044 | -0.00649018 | 0.00241273 | 0.007145766 | 0.562059 | 8 |
| Brain - Cerebellar Hemisphere | rs57902139 | -0.00528904 | 0.00226444 | 0.01950708 | 0.1057401 | 3 |
| Small Intestine - Terminal Ileum | rs950147 | 0.00578385 | 0.00420475 | 0.1689607 | 0.08105564 | 4 |
| Stomach | rs77195211 | -0.00289872 | 0.00211281 | 0.1700708 | 0.477549 | 3 |
| Brain - Nucleus accumbens (basal ganglia) | rs115410750 | -0.00357672 | 0.00265489 | 0.1779088 | NA | NA |
| Adipose - Visceral (Omentum) | rs7620618 | 0.00155944 | 0.00399351 | 0.6961715 | 0.7205832 | 10 |

| **Supplementary Table 9: A cross-tissue evaluation of the association between *SGK223* expression**  **and thyroid disease** |  |  |  |  |  |  |
| --- | --- | --- | --- | --- | --- | --- |
| *Associations between SGK223 expressed in various tissues and thyroid disease in the UK Biobank (defined as self-reported hypothyroidism or*  *myxoedema)* | | | | | | |

| **Tissue** | **Lead eQTL** | **MR Beta** | **MR SE** | **MR P** | **HEIDI P** | **HEIDI nSNPs** |
| --- | --- | --- | --- | --- | --- | --- |
| Thyroid | rs1081975 | -0.00566851 | 0.000966104 | 4.43E-09 | 0.00529054 | 20 |
| Nerve - Tibial | rs1081975 | -0.00485317 | 0.000828051 | 4.60E-09 | 0.1197053 | 20 |
| Skin - Sun Exposed (Lower leg) | rs1873914 | -0.00710954 | 0.00122868 | 7.19E-09 | 0.01349034 | 20 |
| Lung | rs1081975 | -0.0108735 | 0.00194004 | 2.09E-08 | 0.00179813 | 20 |
| Artery - Tibial | rs1873914 | -0.00890642 | 0.00165548 | 7.45E-08 | 0.00050903 | 20 |
| Skin - Not Sun Exposed (Suprapubic) | rs10876864 | -0.00925724 | 0.00173179 | 9.02E-08 | 0.0569747 | 20 |
| Pancreas | rs1081975 | -0.00583751 | 0.00109653 | 1.02E-07 | 0.1595438 | 20 |
| Esophagus - Muscularis | rs1873914 | -0.00794028 | 0.00150869 | 1.42E-07 | 0.07059386 | 20 |
| Brain - Cerebellum | rs1873914 | -0.00510149 | 0.000976793 | 1.76E-07 | 0.08591718 | 20 |
| Artery - Aorta | rs1131017 | -0.0104241 | 0.00203078 | 2.85E-07 | 0.00128434 | 20 |
| Testis | rs1131017 | -0.00854408 | 0.00168789 | 4.15E-07 | 0.01532598 | 20 |
| Spleen | rs705699 | -0.00486107 | 0.000960464 | 4.17E-07 | 0.2212249 | 20 |
| Esophagus - Gastroesophageal Junction | rs1873914 | -0.00736127 | 0.00147124 | 5.63E-07 | 0.3556749 | 20 |
| Colon - Transverse | rs10876870 | -0.00896876 | 0.0018195 | 8.25E-07 | 0.0723682 | 20 |
| Whole Blood | rs1873914 | -0.0148109 | 0.00301657 | 9.12E-07 | 0.2726821 | 20 |
| Pituitary | rs10876870 | -0.0060906 | 0.00124819 | 1.06E-06 | 0.00982821 | 20 |
| Stomach | rs1081975 | -0.00943263 | 0.00195377 | 1.38E-06 | 0.06882311 | 15 |
| Prostate | rs1081975 | -0.00810583 | 0.00168308 | 1.46E-06 | 0.05957294 | 17 |
| Heart - Left Ventricle | rs773114 | -0.0175789 | 0.0036709 | 1.68E-06 | 0.05913912 | 20 |
| Colon - Sigmoid | rs1131017 | -0.00930377 | 0.00196117 | 2.10E-06 | 0.2407559 | 20 |
| Heart - Atrial Appendage | rs10876864 | -0.0162633 | 0.0034474 | 2.39E-06 | 0.00043028 | 20 |
| Small Intestine - Terminal Ileum | rs1081975 | -0.00714872 | 0.00156466 | 4.90E-06 | 0.0316765 | 16 |
| Brain - Cerebellar Hemisphere | rs10876864 | -0.00670471 | 0.00150447 | 8.33E-06 | 0.1742183 | 20 |
| Brain - Caudate (basal ganglia) | rs10876864 | -0.00895714 | 0.002019 | 9.15E-06 | 0.00128948 | 20 |
| Esophagus - Mucosa | rs773114 | -0.0145824 | 0.00336922 | 1.50E-05 | 0.01919002 | 11 |
| Brain - Hippocampus | rs10876864 | -0.00647301 | 0.00149962 | 1.59E-05 | 0.00484735 | 16 |
| Brain - Cortex | rs705699 | -0.00827558 | 0.00192144 | 1.66E-05 | 0.00543265 | 20 |
| Brain - Putamen (basal ganglia) | rs1081975 | -0.00828305 | 0.00193052 | 1.78E-05 | 0.00692559 | 13 |
| Adipose - Subcutaneous | rs10876864 | -0.0183974 | 0.00437605 | 2.62E-05 | 0.00099598 | 20 |
| Brain - Hypothalamus | rs1131017 | -0.00672932 | 0.00163032 | 3.67E-05 | 0.6427532 | 14 |
| Breast - Mammary Tissue | rs1081975 | -0.0146425 | 0.00356464 | 4.00E-05 | 0.00934032 | 20 |
| Muscle - Skeletal | rs2271194 | -0.0351376 | 0.00870677 | 5.44E-05 | 0.4362881 | 14 |
| Ovary | rs1081975 | -0.00887555 | 0.00220961 | 5.90E-05 | 0.04190198 | 14 |
| Brain - Anterior cingulate cortex (BA24) | rs1131017 | -0.0087202 | 0.0021933 | 7.01E-05 | 0.1981342 | 8 |
| Brain - Nucleus accumbens (basal ganglia) | rs10876864 | -0.00807362 | 0.00204743 | 8.04E-05 | 0.00106018 | 20 |
| Brain - Amygdala | rs1131017 | -0.00678094 | 0.00172477 | 8.44E-05 | 0.4429095 | 10 |
| Liver | rs1873914 | -0.0134857 | 0.00375406 | 0.000328 | 0.6213893 | 7 |
| Vagina | rs773125 | -0.00776292 | 0.00217623 | 0.000361 | 0.1601202 | 5 |
| Adrenal Gland | rs7312770 | -0.0134361 | 0.00379703 | 0.000402 | 0.201777 | 6 |
| Adipose - Visceral (Omentum) | rs773114 | -0.0251157 | 0.00719555 | 0.000482 | 0.5756708 | 4 |
| Cells - EBV-transformed lymphocytes | rs705708 | -0.0111162 | 0.00348103 | 0.001406 | 0.5135574 | 3 |
| Artery - Coronary | rs10876942 | -0.000930377 | 0.00222981 | 0.6765 | 0.4246375 | 3 |
| Brain - Frontal Cortex (BA9) | rs61939624 | -0.000166202 | 0.00058062 | 0.774687 | NA | NA |
| Cells - Transformed fibroblasts | rs79563232 | 0.00016368 | 0.00247504 | 0.947273 | NA | NA |

| **Supplementary Table 10: A cross-tissue evaluation of the association between *RNASET2* expression**  **and thyroid disease** |  |  |  |  |  |  |
| --- | --- | --- | --- | --- | --- | --- |
| *Associations between RNASET2 expressed in various tissues and thyroid disease in the UK Biobank (defined as self-reported hypothyroidism or*  *myxoedema)* | | | | | | |

| **Tissue** | **Lead eQTL** | **MR Beta** | **MR SE** | **MR P** | **HEIDI P** | **HEIDI nSNPs** |
| --- | --- | --- | --- | --- | --- | --- |
| Esophagus - Mucosa | rs1130033 | -0.00767221 | 0.00091808 | 6.45E-17 | 0.0962218 | 20 |
| Whole Blood | rs434093 | -0.0097072 | 0.00123727 | 4.31E-15 | 0.0007558 | 20 |
| Cells - Transformed fibroblasts | rs377753 | 0.0058519 | 0.00077009 | 2.98E-14 | 0.0069325 | 20 |
| Skin - Sun Exposed (Lower leg) | rs377232 | -0.0068316 | 0.00089902 | 2.99E-14 | 0.0262752 | 20 |
| Skin - Not Sun Exposed (Suprapubic) | rs377232 | -0.00787656 | 0.00113711 | 4.30E-12 | 0.0174936 | 20 |
| Lung | rs444988 | -0.011646 | 0.00172234 | 1.36E-11 | 0.0464683 | 20 |
| Brain - Cerebellum | rs2757041 | -0.00766933 | 0.00118397 | 9.32E-11 | 0.3113959 | 20 |
| Nerve - Tibial | rs398278 | -0.013689 | 0.00217704 | 3.22E-10 | 0.1025014 | 20 |
| Thyroid | rs1811120 | -0.0216491 | 0.00356195 | 1.22E-09 | 0.0566755 | 20 |
| Pancreas | rs377232 | -0.0139814 | 0.00253208 | 3.36E-08 | 0.3496009 | 20 |
| Adipose - Visceral (Omentum) | rs3798307 | -0.0160056 | 0.00298576 | 8.29E-08 | 0.0446531 | 20 |
| Adipose - Subcutaneous | rs398278 | -0.0270234 | 0.00508631 | 1.08E-07 | 0.0344733 | 20 |
| Brain - Cerebellar Hemisphere | rs398278 | -0.00778497 | 0.00147144 | 1.22E-07 | 0.1664709 | 20 |
| Artery - Tibial | rs3756838 | -0.0257034 | 0.00533179 | 1.43E-06 | 0.2136849 | 20 |
| Vagina | rs4710149 | -0.010689 | 0.00221731 | 1.43E-06 | 0.3442447 | 11 |
| Minor Salivary Gland | rs2247315 | -0.0198882 | 0.00444581 | 7.70E-06 | 0.8985922 | 4 |
| Muscle - Skeletal | rs2769352 | -0.0305634 | 0.00683686 | 7.81E-06 | 0.1816258 | 14 |
| Heart - Atrial Appendage | rs62436765 | -0.0179447 | 0.00415171 | 1.54E-05 | 0.0506773 | 20 |
| Spleen | rs57444856 | -0.0141762 | 0.00332513 | 2.01E-05 | 0.6124955 | 9 |
| Brain - Nucleus accumbens (basal ganglia) | rs398278 | -0.0148683 | 0.00352025 | 2.40E-05 | 0.0999799 | 10 |
| Small Intestine - Terminal Ileum | rs3798307 | -0.0155714 | 0.00370925 | 2.69E-05 | 0.2562698 | 18 |
| Esophagus - Gastroesophageal Junction | rs2236312 | -0.0162916 | 0.00389652 | 2.90E-05 | 0.6341917 | 14 |
| Esophagus - Muscularis | rs2236312 | -0.0261251 | 0.00636015 | 4.00E-05 | 0.3698008 | 17 |
| Cells - EBV-transformed lymphocytes | rs7772112 | -0.0127073 | 0.0031068 | 4.31E-05 | 0.2770883 | 7 |
| Colon - Transverse | rs3756838 | -0.0293269 | 0.00726919 | 5.47E-05 | 0.1819284 | 13 |
| Artery - Coronary | rs6918286 | -0.0169075 | 0.00422645 | 6.32E-05 | 0.1937672 | 20 |
| Brain - Cortex | rs162298 | -0.011641 | 0.00299466 | 0.0001014 | 0.214334 | 7 |
| Stomach | rs57444856 | -0.0202693 | 0.00530757 | 0.000134 | 0.3644339 | 11 |
| Brain - Substantia nigra | rs162295 | -0.0111937 | 0.00304247 | 0.000234 | 0.0539658 | 15 |
| Prostate | rs12526548 | -0.0147947 | 0.00413886 | 0.0003508 | 0.2623651 | 4 |
| Brain - Hippocampus | rs13200640 | 0.00361957 | 0.00255748 | 0.156985 | NA | NA |
| Heart - Left Ventricle | rs117693715 | -0.00291589 | 0.00300167 | 0.3313388 | 0.209584 | 4 |
| Colon - Sigmoid | rs77739713 | -0.00227701 | 0.00296638 | 0.442721 | NA | NA |
| Testis | rs73041490 | 0.00118385 | 0.00229346 | 0.6057259 | 0.8270645 | 7 |

| **Supplementary Table 11: A comparison of associations with thyroid disease**  **across 48 tissues** |  |  |  |  |
| --- | --- | --- | --- | --- |
| *Number of associations with thyroid disease across each tissue in GTEx (excluding HLA region)* | | | | |

| **Tissue** | **Number of associations** | **Sample size (GTEx)** |
| --- | --- | --- |
| Adipose Subcutaneous | 4 | 385 |
| Adipose Visceral Omentum | 0 | 313 |
| Adrenal Gland | 2 | 175 |
| Artery Aorta | 6 | 267 |
| Artery Coronary | 0 | 152 |
| Artery Tibial | 8 | 388 |
| Brain Amygdala | 4 | 88 |
| Brain Anterior cingulate cortex BA24 | 2 | 109 |
| Brain Caudate basal ganglia | 4 | 144 |
| Brain Cerebellar Hemisphere | 8 | 125 |
| Brain Cerebellum | 12 | 154 |
| Brain Cortex | 6 | 136 |
| Brain Frontal Cortex BA9 | 6 | 118 |
| Brain Hippocampus | 4 | 111 |
| Brain Hypothalamus | 4 | 108 |
| Brain Nucleus accumbens basal ganglia | 2 | 130 |
| Brain Putamen basal ganglia | 4 | 111 |
| Brain Spinal cord cervical c-1 | 0 | 83 |
| Brain Substantia nigra | 4 | 80 |
| Breast Mammary Tissue | 0 | 251 |
| Cells EBV-transformed lymphocytes | 2 | 117 |
| Cells Transformed fibroblasts | 6 | 300 |
| Colon Sigmoid | 6 | 203 |
| Colon Transverse | 8 | 246 |
| Esophagus Gastroesophageal Junction | 10 | 213 |
| Esophagus Mucosa | 14 | 358 |
| Esophagus Muscularis | 6 | 335 |
| Heart Atrial Appendage | 0 | 264 |
| Heart Left Ventricle | 2 | 272 |
| Liver | 2 | 153 |
| Lung | 3 | 383 |
| Minor Salivary Gland | 2 | 85 |
| Muscle Skeletal | 4 | 491 |
| Nerve Tibial | 4 | 361 |
| Ovary | 2 | 122 |
| Pancreas | 3 | 220 |
| Pituitary | 1 | 157 |
| Prostate | 1 | 132 |
| Skin Not Sun Exposed Suprapubic | 3 | 335 |
| Skin Sun Exposed Lower leg | 5 | 414 |
| Small Intestine Terminal Ileum | 0 | 122 |
| Spleen | 4 | 146 |
| Stomach | 2 | 237 |
| Testis | 2 | 225 |
| Thyroid | 15 | 399 |
| Uterus | 1 | 101 |
| Vagina | 3 | 106 |
| Whole Blood | 3 | 369 |

| **Supplementary Table 12: Comparing the number of genes which could be**  **instrumented across each tissue compared with their sample size** |
| --- |
| *Number of genes instrumented is based on having at least 1 expression quantitative trait loci with P < 5 x 10-08.* |

| **Tissue** | **Number of genes** | **Sample size (GTEx)** |
| --- | --- | --- |
| Adipose Subcutaneous | 6317 | 385 |
| Adipose Visceral Omentum | 4536 | 313 |
| Adrenal Gland | 2952 | 175 |
| Artery Aorta | 4759 | 267 |
| Artery Coronary | 2005 | 152 |
| Artery Tibial | 6380 | 388 |
| Brain Amygdala | 876 | 88 |
| Brain Anterior cingulate cortex BA24 | 1533 | 109 |
| Brain Caudate basal ganglia | 2255 | 144 |
| Brain Cerebellar Hemisphere | 2829 | 125 |
| Brain Cerebellum | 4050 | 154 |
| Brain Cortex | 2518 | 136 |
| Brain Frontal Cortex BA9 | 1913 | 118 |
| Brain Hippocampus | 1227 | 111 |
| Brain Hypothalamus | 1291 | 108 |
| Brain Nucleus accumbens basal ganglia | 1956 | 130 |
| Brain Putamen basal ganglia | 1586 | 111 |
| Brain Spinal cord cervical c-1 | 1012 | 83 |
| Brain Substantia nigra | 726 | 80 |
| Breast Mammary Tissue | 3549 | 251 |
| Cells EBV-transformed lymphocytes | 1650 | 117 |
| Cells Transformed fibroblasts | 5628 | 300 |
| Colon Sigmoid | 3350 | 203 |
| Colon Transverse | 3848 | 246 |
| Esophagus Gastroesophageal Junction | 3473 | 213 |
| Esophagus Mucosa | 6244 | 358 |
| Esophagus Muscularis | 5876 | 335 |
| Heart Atrial Appendage | 3960 | 264 |
| Heart Left Ventricle | 3631 | 272 |
| Liver | 1832 | 153 |
| Lung | 5968 | 383 |
| Minor Salivary Gland | 924 | 85 |
| Muscle Skeletal | 5663 | 491 |
| Nerve Tibial | 7583 | 361 |
| Ovary | 1628 | 122 |
| Pancreas | 3692 | 220 |
| Pituitary | 2766 | 157 |
| Prostate | 1600 | 132 |
| Skin Not Sun Exposed Suprapubic | 5571 | 335 |
| Skin Sun Exposed Lower leg | 6914 | 414 |
| Small Intestine Terminal Ileum | 1696 | 122 |
| Spleen | 2951 | 146 |
| Stomach | 3095 | 237 |
| Testis | 7008 | 225 |
| Thyroid | 7864 | 399 |
| Uterus | 1087 | 101 |
| Vagina | 1001 | 106 |
| Whole Blood | 4734 | 369 |

| **Supplementary Table 13: Comparing the number of unique genes**  **across tissues which can be instrumented** |  |  |
| --- | --- | --- |
| *Number of genes instrumented is based on having at least 1 expression quantiative trait loci with*  *P < 5 x 10-08 (not found in any other tissue)* | | |

| **Tissue** | **Number of genes** | **Sample size (GTEx)** |
| --- | --- | --- |
| Adipose Subcutaneous | 31 | 385 |
| Adipose Visceral Omentum | 15 | 313 |
| Adrenal Gland | 23 | 175 |
| Artery Aorta | 14 | 267 |
| Artery Coronary | NA | 152 |
| Artery Tibial | 42 | 388 |
| Brain Amygdala | 1 | 88 |
| Brain Anterior cingulate cortex BA24 | 3 | 109 |
| Brain Caudate basal ganglia | 7 | 144 |
| Brain Cerebellar Hemisphere | 12 | 125 |
| Brain Cerebellum | 41 | 154 |
| Brain Cortex | 13 | 136 |
| Brain Frontal Cortex BA9 | 9 | 118 |
| Brain Hippocampus | 4 | 111 |
| Brain Hypothalamus | 8 | 108 |
| Brain Nucleus accumbens basal ganglia | 18 | 130 |
| Brain Putamen basal ganglia | 2 | 111 |
| Brain Spinal cord cervical c-1 | 9 | 83 |
| Brain Substantia nigra | 5 | 80 |
| Breast Mammary Tissue | 4 | 251 |
| Cells EBV-transformed lymphocytes | 20 | 117 |
| Cells Transformed fibroblasts | 95 | 300 |
| Colon Sigmoid | 10 | 203 |
| Colon Transverse | 17 | 246 |
| Esophagus Gastroesophageal Junction | 5 | 213 |
| Esophagus Mucosa | 119 | 358 |
| Esophagus Muscularis | 22 | 335 |
| Heart Atrial Appendage | 14 | 264 |
| Heart Left Ventricle | 20 | 272 |
| Liver | 23 | 153 |
| Lung | 41 | 383 |
| Minor Salivary Gland | 3 | 85 |
| Muscle Skeletal | 127 | 491 |
| Nerve Tibial | 104 | 361 |
| Ovary | 7 | 122 |
| Pancreas | 57 | 220 |
| Pituitary | 12 | 157 |
| Prostate | 5 | 132 |
| Skin Not Sun Exposed Suprapubic | 17 | 335 |
| Skin Sun Exposed Lower leg | 66 | 414 |
| Small Intestine Terminal Ileum | 11 | 122 |
| Spleen | 13 | 146 |
| Stomach | 16 | 237 |
| Testis | 407 | 225 |
| Thyroid | NA | 399 |
| Uterus | 2 | 101 |
| Vagina | 5 | 106 |
| Whole Blood | 148 | 369 |

**Figures**

**Supplementary Figure 1 - Box plot portraying the correlation in our atlas between the number of traits that genes are associated with across number of diverse tissue types**

**
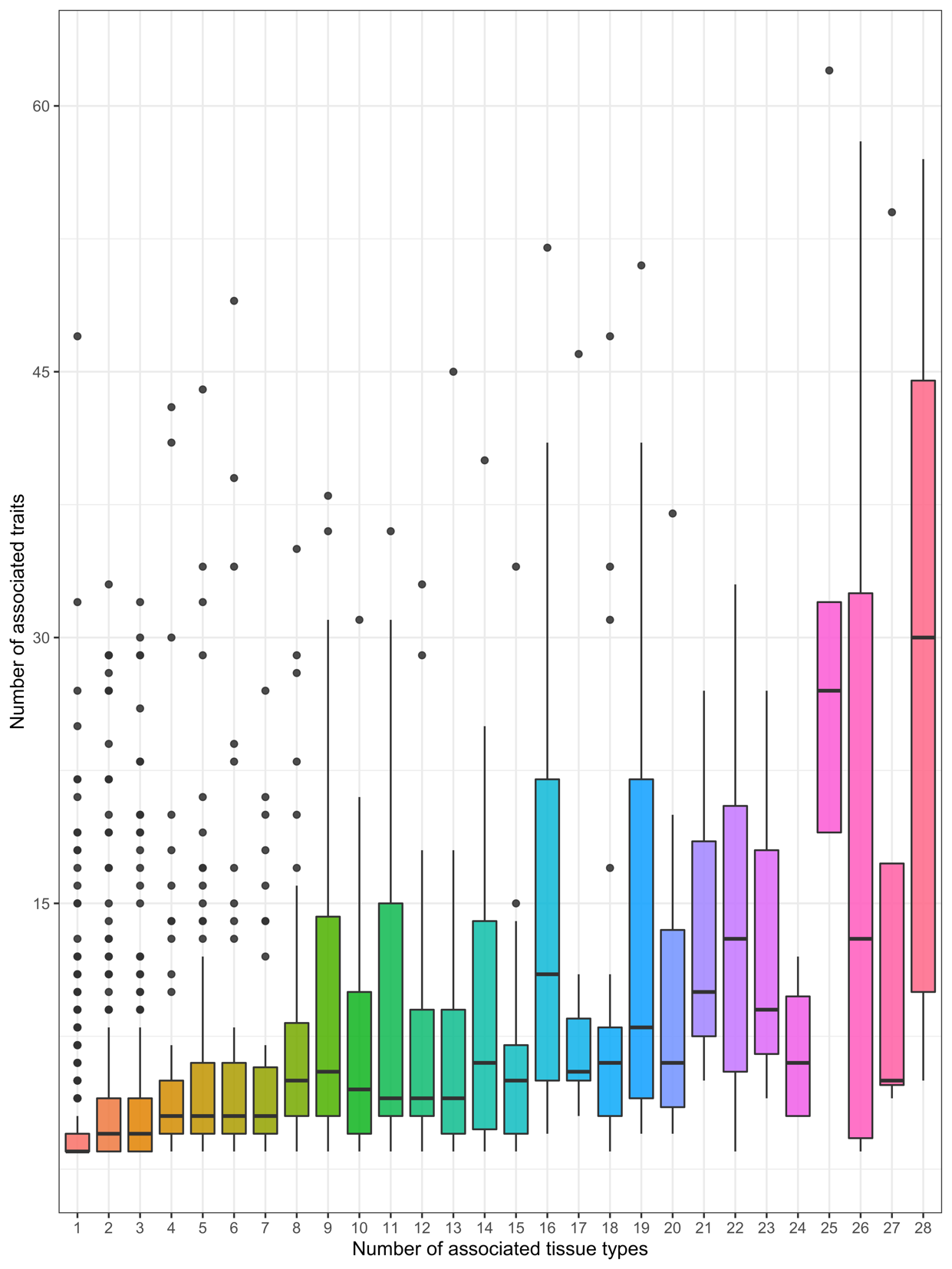
**

**Supplementary Figure 2 – Clustering based of Euclidean distance between tissue types in our results**

**
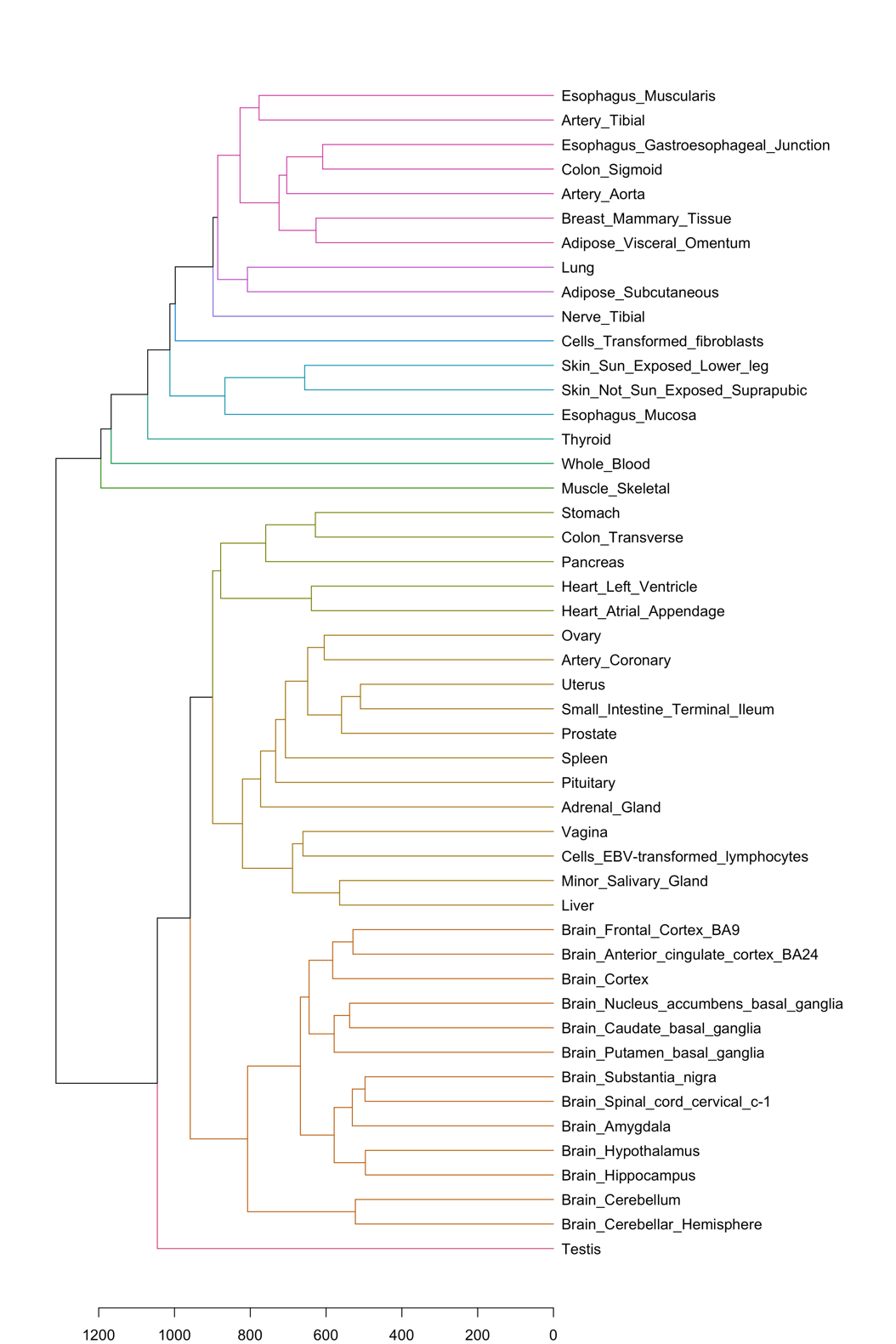
**

**Supplementary Figure 3 – Expression of *RPS26* across all tissue types in GTEx v7**

**
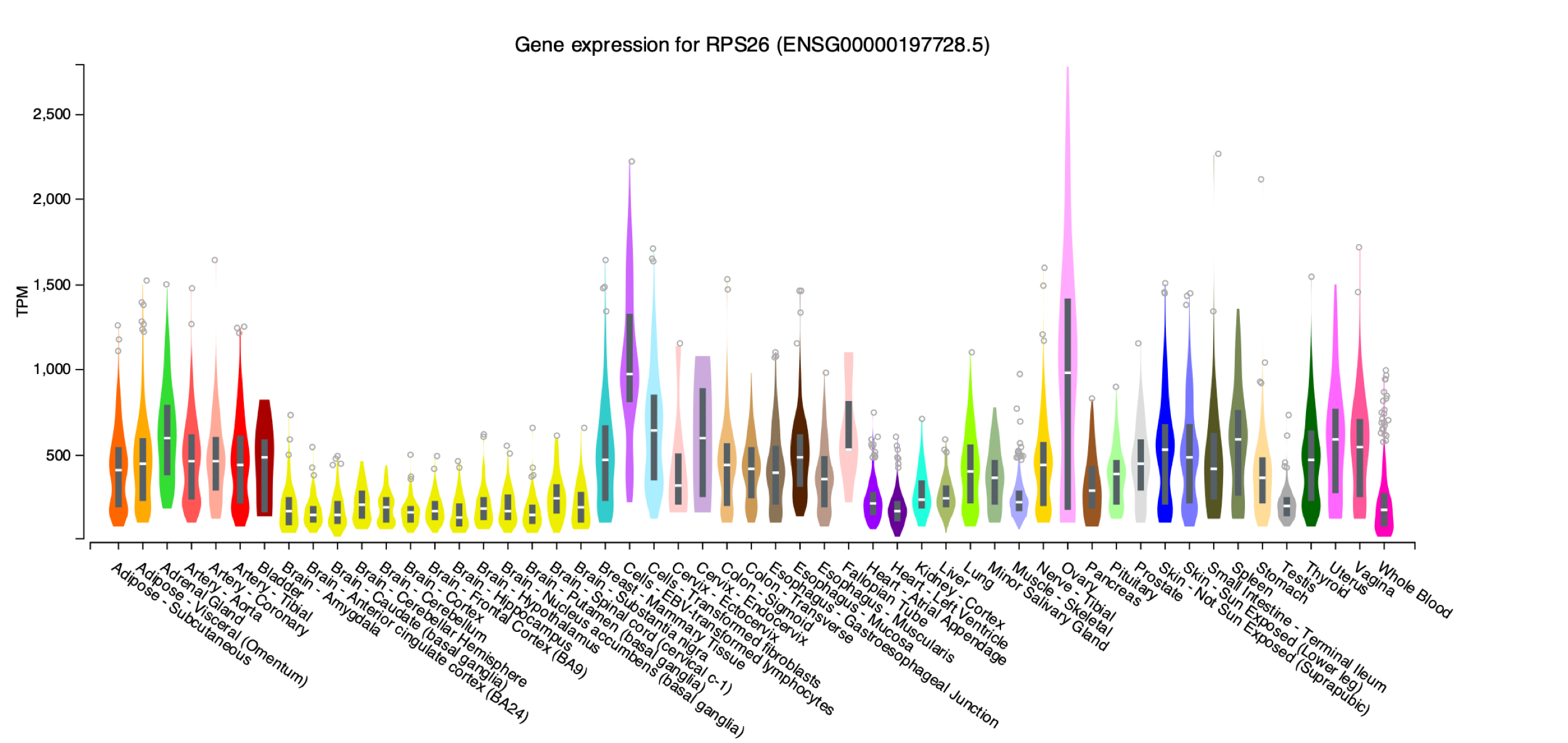
**

**Supplementary Figure 4 – Locus Zoom plot for rs7500448 association with diastolic blood pressure**

**Supplementary Figure 5 – Locus Zoom plot for rs7500448 association with *CDH13* expression**

**Supplementary Figure 6 – Locus Zoom plot for rs7500448 association with *RP11-483P21.3* expression**

**Supplementary Figure 7 – Locus Zoom plot for rs7500448 association with *RP11-483P21.2* expression**

**Supplementary Figure 8 – Locus Zoom plot for rs1706003 association with diastolic blood pressure**

**Supplementary Figure 9 – Locus Zoom plot for rs1706003 association with *ATP13A3* expression**

**Supplementary Figure 10 – Locus Zoom plot for rs1706003 association with *LINC00884* expression**

**Supplementary Figure 11 – Locus Zoom plot for rs1706003 association with *TMEM-AS1* expression**

**Supplementary Figure 12 – Locus Zoom plot for rs1706003 association with *LSG1* expression**

**Supplementary Figure 13 – Locus Zoom plot for rs1706003 association with *TMEM44* expression**

**Supplementary Note 1:**

For the selected few genes with more than 1 instrument identified in the analysis between thyroid-derived gene expression and thyroid disease (n=285), we also analysed effects using a multiple SNP MR approach (known as the inverse variance weighted method^1^). There was evidence of association for 13 loci (based on P<0.05/285), although only 5 of these were not located in the HLA region of the genome (Supplementary Table 24). We note that this analysis suggests that future analyses using multi-SNP MR approaches may provide additional power to detect loci over single SNP approaches.

**References:**

1. Burgess, S., Butterworth, A. & Thompson, S.G. Mendelian randomization analysis with multiple genetic variants using summarized data. *Genet Epidemiol* **37**, 658-65 (2013).
